# Diltiazem Dosing Strategies in the Management of Atrial Fibrillation With Rapid Ventricular Rate

**DOI:** 10.7759/cureus.18829

**Published:** 2021-10-16

**Authors:** James Bishop, Ghufraan Akram

**Affiliations:** 1 Emergency Medicine, Ascension Providence Hospital-Southfield, Southfield, USA; 2 Emergency Medicine, Michigan State University College of Human Medicine, East Lansing, USA

**Keywords:** emergency department, heart rate control, diltiazem, rapid ventricular response, atrial fibrillation

## Abstract

Introduction

Diltiazem is commonly used for the management of atrial fibrillation (AFIB) with rapid ventricular rate (RVR) in the emergency department (ED). Conflicting studies comparing the efficacy of diltiazem have led to various dosing strategies. The objective of this study was to investigate diltiazem administration in a community ED and determine the effect of varying doses on heart rate (HR) control, systolic blood pressure (SBP) and diastolic blood pressure (DBP).

Methods

This is a retrospective, single-center study of adult patients treated with diltiazem for AFIB-RVR in the ED between January 1 and December 31, 2019. Inclusion criteria included pretreatment HR > 120 beats per minute (bpm). Patients were administered diltiazem at the discretion of the ED physician. Primary endpoint was time to achieve HR < 100 bpm after diltiazem. Secondary endpoints included mean weight-based dose of diltiazem, percentage of patients achieving HR < 100 bpm within 240 minutes of diltiazem, nadir SBP and nadir DBP.

Results

Ninety-nine patients were included in the study. Seventy-two percent of patients received ≤ 10 mg diltiazem bolus. Mean weight-based dose of diltiazem bolus was 0.13 mg/kg. Mean time to achieve HR < 100 bpm was 270 minutes for the entire cohort. Patients treated with ≥ 0.13 mg/kg diltiazem achieved an HR < 100 bpm at a mean time of 169 minutes compared to 318 minutes for < 0.13 mg/kg (p = 0.0107). HR control was achieved in 61% of patients who received ≥ 0.13 mg/kg compared to 36% of patients who received < 0.13 mg/kg diltiazem (p = 0.0213). No patients discontinued diltiazem for hypotension or bradycardia. The lowest recorded SBP and DBP within 240 minutes of diltiazem were 90 mmHg and 47 mmHg, respectively. There was no significant difference in the lowest SBP and DBP for patients who received < 0.13 mg/kg compared to ≥ 0.13 mg/mg diltiazem.

Conclusion

The majority of patients with AFIB RVR received a 10 mg non-weight-based diltiazem bolus dose in the ED. Diltiazem bolus dosing ≥ 0.13 mg/kg was associated with significantly improved times to achieve HR control compared to < 0.13 mg/kg and was not associated with hypotension or bradycardia.

## Introduction

Atrial fibrillation (AFIB) is a commonly encountered arrhythmia in the hospital setting and the primary diagnosis for over 600,000 emergency department (ED) visits and 460,000 hospitalizations in the United States, each year [1]. AFIB is a risk factor for stroke by predisposing to thrombus formation, may exacerbate heart failure due to loss of effective atrial contraction, and may cause tachycardia-induced cardiomyopathy when ventricular rate is not controlled for prolonged periods of time [2,3]. To prevent complications and relieve patient symptoms, ventricular rate should be controlled in these patients. 

For many ED physicians, diltiazem has become the preferred agent to achieve rate control of AFIB with rapid ventricular rate (AFIB-RVR). Diltiazem is effective in treating AFIB-RVR by slowing conduction through the atrioventricular node (AV) and by prolonging AV node refractoriness. When compared head-to-head with metoprolol or digoxin, diltiazem bolus was superior in achieving ventricular rate control at 30 minutes post-administration [4,5]. According to the American College of Cardiology Foundation, American Heart Association and European Society of Cardiology (ACC/AHA/ESC) 2006 Guidelines for the Management of Patients with Atrial Fibrillation, the recommended diltiazem dosing should be 0.25 mg/kg bolus, followed by an intravenous (IV) drip of 5-15 mg/hour [6]. Recent studies have suggested that low-dose diltiazem dosing strategies may be as effective as standard dosing for rate control in AFIB, thus prompting healthcare providers to prescribe < 0.25 mg/kg diltiazem for AFIV-RVR [7,8]. In our experience, diltiazem dosing strategies vary among providers. This study investigated diltiazem dosing strategies administered to patients who present with AFIB-RVR in a suburban ED and compared doses with time to achieve ventricular rate control.

## Materials and methods

This was a retrospective, observational, single-center study of adult patients who were treated with diltiazem for AFIB-RVR in an ED from January 1, 2019, to December 31, 2019. The study was conducted at a community hospital ED with approximately 43,000 annual ED visits. Inclusion criteria included the following: patients ≥ 18 years of age who presented to the ED in AFIB with an HR > 120 beats per minute (bpm) and received diltiazem in the ED. Diltiazem dose and route selection were given at the discretion of the ED physician. Patients were excluded if they presented with fever (≥ 38° C), suspected alcohol or drug withdrawal, received cardioversion or other rate control or anti-arrhythmic medications in the ED setting before diltiazem administration, or if there was no documentation of blood pressure or HR after the study intervention. The primary endpoint was defined as time to achieve ventricular rate < 100 bpm after diltiazem administration. Secondary endpoints included mean dose of diltiazem, percentage of patients achieving HR < 100 bpm within 240 minutes of initial diltiazem administration, nadir SPB and DPB within 240 minutes after diltiazem dosing. A clinical endpoint of 240 minutes was selected to give time for diltiazem to have a therapeutic effect and a reasonable time for the ED to determine disposition.

The safety endpoint was defined as an acute adverse event within the first 240 minutes of diltiazem treatment in the form of bradycardia (HR < 50 bpm), hypotension (SBP < 90 mmHg), respiratory failure requiring noninvasive positive pressure ventilation or intubation, vasopressor administration or death. The study was approved by the local institutional review board.

For those patients who meet the inclusion and exclusion criteria, data were extracted from the medical record chart and used for study analysis. Baseline demographics included patient’s age, gender, weight, HR, SBP, DBP and home medications taken for rate control. The total diltiazem dose delivered in ED and route of administration (IV bolus, IV continuous infusion, IV bolus and continuous infusion) was captured. The data were analyzed using Microsoft Excel (Microsoft Corporation, Redmond, WA) and GraphPad Prism (GraphPad Software, San Diego, CA). Quantitative variables were expressed as mean and standard deviation or standard error of the mean, and categorical data were reported as number and percentages (%). Variables were evaluated with X^2^ or Fisher’s exact test and continuous variables evaluated using Student’s t-test. Univariate and multivariate analysis was conducted to assess the association of patient and treatment variables with time to achieve ventricular rate control. Statistical significance was considered significant if p < 0.05.

## Results

From January 1 through December 31, 2019, 123 adult patients who received diltiazem in the ED for AFIB-RVR were identified. Of these patients, 24 patients were excluded due to either alcohol withdrawal, fever, administration of other rate control or anti-arrhythmic medications in the ED setting before diltiazem administration, or cardioversion. Complete data were available for 99 patients. Patient characteristics are shown in Table 1. The mean age was 74 years (range: 30-99 years), mean weight was 88 kg (range: 45-170 kg), 57% were female and 43% were male gender. The mean baseline HR was 146 bpm (range: 120-197 bpm), mean baseline SBP was 132 mmHg (range: 90-181 mmHg) and mean baseline DBP was 89 mmHg (range: 50-143 mmHg).

**Table 1:** Baseline patient characteristics bpm: beats per minute, HR: heart rate, SBP: systolic blood pressure, DBP: diastolic blood pressure.

Variable	N
Male (%)	43 (43%)
Female (%)	56 (57%)
Mean age (range), years	74 (30-99)
Mean weight (range), kg	88 (45-170)
Mean HR (range), bpm	146 (120-197)
Mean SBP (range), mmHg	132 (90-181)
Mean DBP (range), mmHg	89 (50-143)
Method of diltiazem treatment:	
Bolus only (%)	54 (55%)
Continuous infusion only (%)	8 (8%)
Bolus + continuous infusion (%)	37 (37%)
Mean diltiazem bolus dose (range), mg	10.73 (5-25)
Mean diltiazem bolus dose (range), mg/kg	0.13 (0.39-0.292)
Diltiazem bolus dose:	
5.0 mg (%)	17 (19%)
10 mg (%)	55 (60%)
15 mg (%)	9 (10%)
18 mg (%)	2 (2%)
20 mg (%)	6 (7%)
25 mg (%)	2 (2%)
Mean diltiazem continuous infusion dose (range), mg/hour	5.6 (5-15)
Diltiazem continuous infusion dose:	
5 mg/hour (%)	40 (89%)
10 mg/hour (%)	4 (9%)
15 mg/hour (%)	1 (2%)
Home medications:	
Beta-blocker (%)	41 (41%)
Calcium channel blocker (%)	34 (34%)
Anti-arrhythmic (%)	11 (11%)

**Figure 1:**
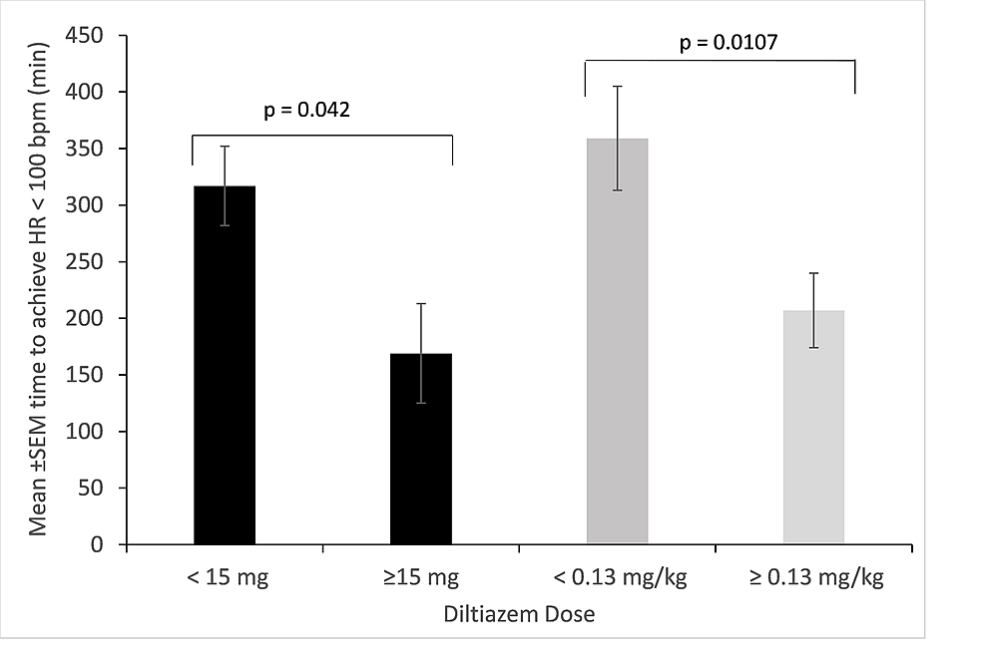
Time to achieve HR < 100 bpm after diltiazem bpm: beats per minute, HR: heart rate, SEM: standard error of the mean.

For all patients, the mean time between the first diltiazem treatment and time to achieve an HR < 100 bpm was 270 minutes (range: 5-2,040 minutes). The mean time to achieve an HR < 100 bpm was 278 minutes (range: 7-781 minutes) for 5 mg, 330 minutes (range: 1-2,040 minutes) for 10 mg, and 169 minutes (range: 3-580 minutes) for ≥ 15 mg bolus diltiazem. There was a statistically significant difference in time to achieve HR control in patients who received diltiazem bolus ≥ 15 mg compared to patients who received < 15 mg (169 minutes vs 318 minutes, respectively; p = 0.042; Figure 1). Increased weight-based diltiazem dose was associated with decreased time to achieve a heart rate < 100 bpm. The mean time to achieve an HR < 100 bpm was 206 ± 32 minutes for patients who received ≥ 0.13 mg/kg diltiazem compared to 359 ± 46 minutes for patients who received < 0.13 mg/kg (p = 0.0107; Figure 1). 

**Figure 2:**
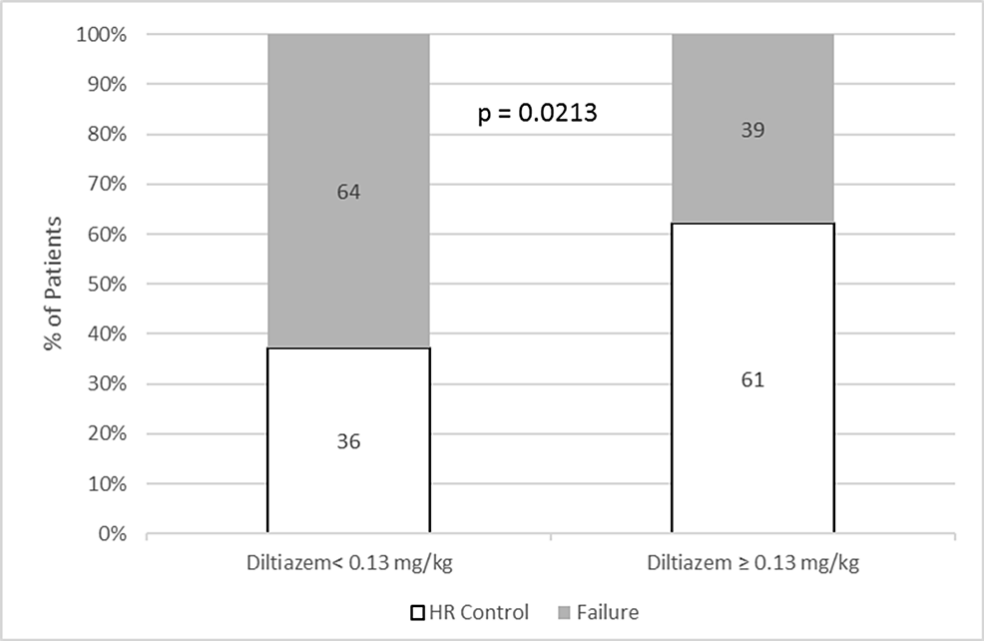
Percentage of patients with controlled HR (< 100 bpm) after diltiazem bpm: beats per minute, HR: heart rate.

For the endpoint of achieving ventricular rate control (HR < 100 bpm) within 240 minutes of initial diltiazem administration, 46% of all patients achieved HR control. When stratified by weight-based low-dose (< 0.13 mg/kg) vs high-dose (≥ 0.13 mg/kg) diltiazem, 61% of patients who received > 0.13 mg/kg diltiazem achieved HR control compared to 36% of patients who received < 0.13 mg/kg (p = 0.0213; Figure 2).

When baseline characteristics of patients who received diltiazem doses < 0.13 mg/kg vs ≥ 0.13 mg/kg were evaluated, weight was statistically different between the two groups (Table 2). Patients who received < 0.13 mg/kg diltiazem had a mean weight of 97 ± 30 kg compared to 74 ± 20 kg for the ≥ 0.13 mg/kg diltiazem group (p < 0.001). There were no statistical differences (all p values > 0.05 between groups) between pretreatment age, heart rate, use of rate controlling home medications or pretreatment SBP or DBP when comparing higher dose (≥ 0.13 mg/kg) to lower dose (< 0.13 mg/kg) males to males or females to females.

**Table 2:** Baseline characteristics of patients treated with < 0.13 mg/kg vs ≥ 0.13 mg/kg bolus diltiazem SD: standard deviation, HR: heart rate, bpm: beats per minute, SBP: systolic blood pressure, DPB: diastolic blood pressure.

Variable	Diltiazem < 0.13 mg/kg Mean ± SD (n = 50)	Diltiazem ≥ 0.13 mg/kg Mean ± SD (n=41)	p-Value
Age, years	72 ± 15	77 ± 12	0.080
Weight, kg	97 ± 30	74 ± 20	< 0.001
HR, bpm	144 ± 21	149 ± 20	0.237
SBP, mmHg	130 ± 19	137 ± 25	0.117
DPB, mmHg	89 ± 16	90 ± 17	0.766

No patients experienced bradycardia, hypotension or death in the ED. The lowest recorded HR within 240 minutes after diltiazem was 70 bpm (range: 70-163 bpm; mean HR: 101.5 bpm; Table 3). The lowest recorded SBP within 240 minutes of diltiazem was 90 mmHg (range: 90-172 mmHg; mean: 116 mmHg) and the lowest recorded DBP within 240 minutes after diltiazem was 47 mmHg (range: 47-114; mean: 74.6 mmHg). When stratified by low dose (< 0.13 mg/kg) and high dose (≥ 0.13 mg/kg), patients who received high-dose diltiazem had significantly lower nadir HR (mean: 97.3 ± 14.1 bpm) compared to patients who received low-dose diltiazem (mean: 105.8 ± 18.0 bpm; p = 0.026; Table 3). No statistically significant differences were observed for SBP or DBP when comparing patients who received < 0.13 mg/kg) vs ≥ 0.13 mg/kg diltiazem.

**Table 3:** Lowest HR, SBP and DBP within 240 minutes after diltiazem bolus administration bpm: beats per minute, HR: heart rate, SBP: systolic blood pressure, DPB: diastolic blood pressure.

Variable	All patients Mean ± SD (n=91)	< 0.13 mg/kg diltiazem Mean ± SD (n = 50)	≥ 0.13 mg/kg diltiazem Mean ± SD (n = 41)	p-Value
HR, bpm	101.5 ± 16.8	105.8 ± 18.0	97.3 ± 14.1	0.026
SBP, mmHg	116.0 ± 17.4	114.5 ± 2.0	116.9 ±3.0	0.48
DBP, mmHg	74.6 ± 14.8	76.9 ± 15.2	71.8 ± 13.7	0.058

## Discussion

Despite established diltiazem dosing guidelines [6], physicians often administer a bolus dose of diltiazem that is lower than the recommended 0.25 mg/kg [7-9]. In a study by Ross et al., the authors reported 56% of the patients treated with diltiazem for AFIB-RVR were given a 10 mg, non-weight-based dose [8]. Lee et al. observed 33% of the patients received a bolus dosing less than the recommended 0.25 mg/kg, and Ward et al. reported 39% of patients in their study did not receive standard, weight-based dosing [7,9]. Similar to these studies, we report 72% of patients received a diltiazem bolus dose ≤ 10 mg and the mean weight-based bolus dose given was 0.13 mg/kg (range: 0.04-0.29 mg/kg).

Physicians treating AFIB-RVR may be influenced to administer lower, non-weight-based diltiazem dosing due to concerns of reported hypotension adverse events. In a study by Dougherty et al., 16% of all patients reported hypotension (SBP < 90 mmHg) after receiving 0.05 to 0.45 mg/kg diltiazem for HR control of supraventricular tachycardia [10]; however, there was no difference in rates of hypotension between the low- and high-dose groups. In a retrospective chart review by Lee et al., the authors investigated HR control and hypotension in patients with AFIB-RVR who received low-dose (≤ 0.2 mg/kg), standard dose (> 0.2 ≤ 0.3 mg/kg) or high-dose (>0.3 mg/kg) diltiazem [7]. The authors defined hypotension as SBP < 90 mmHg or reduction of SBP ≥ 20% from baseline. The study showed that the percentage of patients who achieved a reduction of SPB ≥ 20% from baseline was higher in patients who received high-dose diltiazem; however, there was no statistically significant difference in the rates of SBP < 90 mmHg between low (1.6%), standard (7.2%) and high dose (2.8%). Furthermore, the overall percent reduction of SBP for the low-dose, standard dose and high-dose groups was not statistically different. Multivariate analysis did show a reduction of SBP ≥ 20% from baseline for the lower-dose group compared to the standard dose group, but not for the high-dose group, suggesting that it is still unclear as to the impact of higher doses of diltiazem in relation to hypotension and adverse events. In our study, no patients had an SBP < 90 mmHg within 240 minutes of diltiazem and there was no difference in the lowest reported SBP in patients who received < 0.13 vs ≥ 0.13 mg/kg diltiazem. It is important to note that the highest bolus dose administered in our study was 0.25 mg/kg; therefore, we cannot exclude that diltiazem doses > 0.25 mg/kg are associated with adverse events. Future, prospective studies are warranted to evaluate the role of diltiazem and its association with hypotension and adverse events.

Recent studies have suggested that low-dose, non-weight-based diltiazem may be as effective as standard dosing in achieving ventricular control for AFIB-RVR [7,8]. Ross et al. concluded that 10 mg IV dosing of diltiazem was non-inferior in achieving ventricular rate control compared to weight-based dosing [8]. Lee et al. concluded that the rates of therapeutic response were similar for low-dose, standard dose and high-dose diltiazem [7]. Our study differs from these studies in the definition of ventricular rate control. Ross et al. and Lee et al. included a 20% reduction in HR from baseline within their definition of HR control [7,8]. We defined successful HR control as a post-treatment HR < 100 bpm, which is the definition used by Schreck et al. in their prospective open label study comparing IV diltiazem to IV digoxin [5]. In our patient population, several patients had an initial HR > 190 bpm, with a maximum HR of 197 bpm. A 20% reduction of baseline HR of 197 bpm is 157.6 bpm. This would meet the definition of therapeutic response in the above studies. We argue that this definition of rate control may not be sufficient to prevent the patient from experiencing symptoms or complications such as ischemia, heart failure or organ hypoperfusion. When evaluating reports describing the efficacy of diltiazem dosing strategies, it is imperative for the reader to understand the definition of HR control used in the study, which may have different clinical implications. 

The ability to achieve rate control within 240 minutes has many potential benefits for the patient and treating facility, including increased patient comfort and decreased utilization of physician, nursing and hospital resources. Patients who achieve rate control during their ED visit may be candidates for discharge to home, if appropriately anticoagulated, while those who remain in AFIB-RVR may have prolonged ED length of stay or require hospital admission until HR < 100 bpm is achieved. We report the dosing strategy to achieve timely rate control (HR < 100 bpm) without clinically significant hypotension is at least 0.13 mg/kg diltiazem. Future studies need to be done to investigate the association of weight-based diltiazem, HR control and patient length of stay.

There are several limitations to our study that need to be acknowledged. First, this was a retrospective, single-institution study and therefore may have inherent physician bias. As previously noted, diltiazem dosing and route of administration were non-standardized and were prescribed at the discretion of the ED physician, and this resulted in variations of diltiazem dosing and administration strategies. Documentation of vital signs was not consistent, and repeat vitals were not uniformly collected at every hour. Reporting inconsistencies are not unexpected in a busy ED, where physician and nursing staff resources fluctuate depending on patient volume and acuity of illness. Documentation could have been completed less frequently in patients who were perceived to be more stable. Because of these limitations, larger confirmatory studies are needed to prospectively compare efficacy and safety of weight-based diltiazem dosing strategies to standardize non-weight-based dosing strategies, which may encourage physicians to follow dosing guidelines.

## Conclusions

In our study, patients who presented to the ED with AFIB-RVR achieved successful HR control in less time when treated with weight-based diltiazem bolus ≥ 0.13 mg/kg compared to patients treated with < 0.13 mg/kg. Higher doses of diltiazem were not associated with higher incidence of hypotension or bradycardia. Prospective studies comparing weight-based dosing strategies to non-weight-based diltiazem are warranted.
